# Does Predation Risk Affect Mating Behavior? An Experimental Test in Dumpling Squid (*Euprymna tasmanica*)

**DOI:** 10.1371/journal.pone.0115027

**Published:** 2014-12-31

**Authors:** Amanda M. Franklin, Zoe E. Squires, Devi Stuart-Fox

**Affiliations:** Zoology Department, The University of Melbourne, Melbourne, Victoria 3010, Australia; University of Melbourne, Australia

## Abstract

**Introduction:**

One of the most important trade-offs for many animals is that between survival and reproduction. This is particularly apparent when mating increases the risk of predation, either by increasing conspicuousness, reducing mobility or inhibiting an individual's ability to detect predators. Individuals may mitigate the risk of predation by altering their reproductive behavior (e.g. increasing anti-predator responses to reduce conspicuousness). The degree to which individuals modulate their reproductive behavior in relation to predation risk is difficult to predict because both the optimal investment in current and future reproduction (due to life-history strategies) and level of predation risk may differ between the sexes and among species. Here, we investigate the effect of increased predation risk on the reproductive behavior of dumpling squid (*Euprymna tasmanica*).

**Results:**

Females, but not males, showed a substantial increase in the number of inks (an anti-predator behavior) before mating commenced in the presence of a predator (sand flathead *Platycephalus bassensis*). However, predation risk did not affect copulation duration, the likelihood of mating, female anti-predator behavior during or after mating or male anti-predator behavior at any time.

**Conclusions:**

Inking is a common anti-predator defense in cephalopods, thought to act like a smokescreen, decoy or distraction. Female dumpling squid are probably using this form of defense in response to the increase in predation risk prior to mating. Conversely, males were undeterred by the increase in predation risk. A lack of change in these variables may occur if the benefit of completing mating outweighs the risk of predation. Prioritizing current reproduction, even under predation risk, can occur when the chance of future reproduction is low, there is substantial energetic investment into mating, or the potential fitness payoffs of mating are high.

## Introduction

Many animals face a fundamental trade-off between survival and reproduction. This trade-off has important consequences for the evolution of life-history strategies (e.g. investment into current versus future reproduction), and sexual selection (e.g. degree of choosiness; [1], [2]). The optimal solution to this trade-off will be influenced by both lifetime reproductive opportunities and the risk or frequency of predation. For example, under an increased risk of predation black gobies (*Gobius niger*) refrain from nest building and spawning whereas a closely related species, sand gobies (*Pomatoschistus minutus*), do not alter nest building and spawning behaviors [3]. This difference in response to predation risk may reflect differences in future reproductive opportunities; black gobies can reproduce for several years while sand gobies have only one breeding season [3]. Predation risk also influences differences in reproductive behavior between individuals within species [4], [5]. For example, female Trinidadian guppies (*Poecilia reticulata*) from populations where predation is high will reduce their sexual activity and become less choosy in the presence of a fish predator [6]. Conversely, females from populations where predation is low do not exhibit these behavioral changes in response to a predator [6]. Thus, the degree to which individuals modulate their reproductive behavior in relation to predation risk varies within and between species and is often difficult to predict.

Sexual reproduction can increase predation risk by reducing mobility, increasing conspicuousness due to movement, position or compromised camouflage, or by inhibiting an individual's ability to detect predators [7]–[12]. This increase in risk applies to many components of reproduction including mate search, courtship, copulation, mate-guarding, carrying eggs or young, and parental care [7]–[9], [11], [13]. Individuals can mitigate the potential predation costs of reproduction by adjusting one or more of these components of reproductive behavior when predation risk is high. For example, one strategy in situations of high predation risk is to reduce mating frequency (e.g. pipefish [14]; threespine sticklebacks [15]; fiddler crabs [16]; amphipods [17]; guppies [6]). In other studies individuals mitigate predation risk by reducing the duration or intensity of courtship (e.g. threespine sticklebacks [18]; Lake Eyre dragons [19]; tungara frogs [20]), relaxing choosiness (e.g. amphipods [17]; guppies [6]), reducing or stopping display signals (e.g. tungara frogs [20], [21]), reducing copulation duration (e.g. semiaquatic hemipterans [22]) and/or altering the timing of reproduction (e.g. gobies [23]). By contrast, other studies report no effect of predation risk on the probability or duration of copulation (e.g. milkweed beetles [24]; guppies [25]; wolf spiders [26]). When the effect of predation risk on mating behavior varies between species, this is likely to reflect differences in life history (e.g. probability of future reproduction), ecology (e.g. natural predation levels) and physiology (e.g. time required for successful sperm transfer).

The majority of studies demonstrate that mating pairs are particularly likely to experience increased predation risk due to a combination of increased vulnerability and because they may be specifically targeted; however, the risks may differ between the sexes. For example, burrows of paired crickets are attacked by predators four times more often than those of lone crickets [27]. However, because paired males allow females priority access to the burrow, they experience an almost four-fold increase in the probability of being killed when attacked by a predator compared with lone individuals. In contrast, paired females experience more than a five-fold decrease in predation compared with lone individuals [27]. When future reproductive opportunities are limited, males must only survive long enough to transfer sperm, whereas females must survive long enough to lay eggs or raise offspring. This difference in the interests of the sexes may influence risk taking behavior and responses to predation risk [7], [10], [27], [28]. Thus, even for paired individuals, behavioral responses to predation risk can differ markedly between the sexes.

In order to make generalizations regarding trade-offs between survival and reproduction, it is important to assess the effects of predation risk on mating behavior for each sex, in species with a wide range of ecologies and life-histories. Here we test whether dumpling squid, *Euprymna tasmanica*, modify their mating behavior in response to predation risk. Cephalopods are an understudied but particularly interesting group in which to address this question due to their excellent vision and cognitive ability, generally high levels of multiple mating in both sexes [29]–[32], and, as soft bodied invertebrates, vulnerability to predation. In dumpling squid, both sexes mate multiply within and between reproductive bouts [33] and mating duration is unusually long, lasting up to 3 hours in some mating pairs (86.1±7.6 min (mean ±SEM), range: 48.3–184.2 min; [34]). Males transfer the spermatophore bundle to females within the first five minutes of copulation; however, the long copulation duration is required for the male to successfully evert the spermatophores into the female's sperm storage organ (pers. obs.). During mating, male *E. tasmanica* insert and then enlarge their sperm transfer organ (the hectocotylus) into the female's spermatheca. Removal of the hectocotylus at the end of copulation requires forceful movements by the male (S1 Video; [35]). Furthermore, males use their arms to physically restrain the female for the duration of copulation. These characteristics of mating suggest that males control mating duration. We predicted that current reproductive opportunities may be important for *E. tasmanica* because they are solitary, live for less than one year and are only sexually mature for 80±5.7 days (mean ±SEM; S1 Table) in the laboratory (although some mating may occur before maturity). In captivity, females lay a series of egg clutches (mean  = 4.56±0.34, max  = 13) over an average of 36.41±3.65 days (max  = 121 days; [35]).

**Figure 1:**
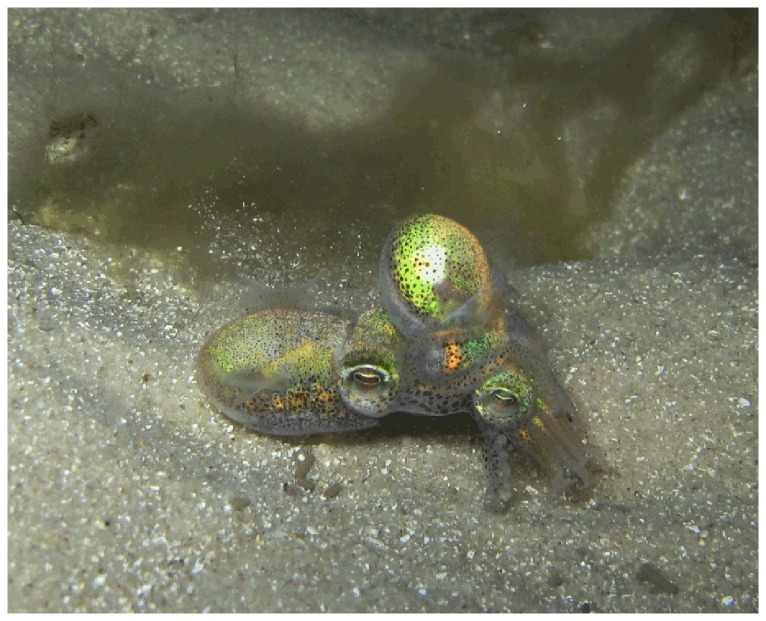
A mating pair of *Euprymna tasmanica* in the field (male left, photo: Zoe Squires).

Predation risk is likely to be an important component of mating decisions for dumpling squid. Individual squid are usually found on the benthos or buried in the sand, but mate in the water column. This makes mating pairs potentially more conspicuous to visual predators than individual squid. In addition, dumpling squid mate in the ‘male-to-female neck’ position (Fig. 1) which exposes the lighter colored underside of the female, arguably making her even more visible. To escape predators, squid can use flee tactics including jets and inks (S1 Video). A jet is when a squid forcefully ejects water through its siphon, propelling it rapidly backward and an ink is when a squid ejects a dark cloud of chemicals. Jetting is a form of locomotion and can be used as a flee response, and inking is used to confuse the receiver. Both behaviors are effective against ambush and cruising predators [36]. However, mating appears to greatly reduce the mobility of the mating pair (the female's siphon which is used for propulsion is restricted by the male) therefore reducing the pair's ability to escape a predatory attack. Furthermore, it is unlikely that pairs are able to separate easily in response to an attack due to the male's enlarged hectocotylus inserted into the female. Dumpling squids' association with the benthos, and the presence of a light organ that can diminish their silhouette from below [37], suggest that benthic predators are a major threat. We tested whether male and female squid alter their likelihood of mating, duration of copulation and anti-predator behaviors prior to, during and after mating in response to a benthic fish predator. We discuss the observed patterns in relation to life history and probable current and future reproductive opportunities.

## Materials and Methods

### Squid culture

We collected mature *Euprymna tasmanica* from May 2010 until February 2011 during multiple shallow (<5 m) night SCUBA dives at Clifton Springs (38°09′18S, 144°34′03E) and St Leonards (38°10′13S, 144°43′11E) in Port Phillip Bay, Victoria, Australia. Squid with gonads visible through the translucent ventral wall were considered mature [38]. Upon capture, squid were transferred to facilities at the Victorian Marine Science Consortium (VMSC) in Queenscliff. Here they were housed individually in round plastic buckets (diameter × height: 20×19 cm, volume  = 6.0 L) with no water flow among holding aquaria. Each holding aquarium contained a layer of sand substrate and a short (diameter × length: 5.5×6.5 cm) length of PVC pipe in which to lay eggs and shelter. Aquarium lights provided a reverse 12:12 hr day/night cycle and all aquaria received a constant flow of aerated, ambient temperature (13–21°C) seawater, pumped directly from Port Phillip Bay. Squid were allowed to acclimate in the lab for at least 14 days. Squid were checked every second day and fed squid *Palaemon* shrimp *ad libitum*. Following this experiment, squid were maintained in the laboratory for use in other experiments.

### Sand flathead culture

Sand flathead, *Platycephalus bassensis*, were used as a predator because they are medium-sized, ambush predators which are found in the same habitat as dumpling squid. We regularly saw flathead lying semi-concealed in the sand during our night-dives for dumpling squid. Furthermore, they are generalist predators, consuming crabs, shrimp, small fish and squid [39]) and we have observed one eat a dumpling squid. We captured sand flathead (*n* = 15) by line using calamari bait in Port Phillip Bay, Victoria, Australia (38°08′03S, 144°48′30E) during July and October 2010. Only fish over 22cm were used to ensure they were large enough to eat dumpling squid. Upon capture, fish were placed in a fish bucket with seawater and transferred to a 1000 L plastic holding tank covered with a shade cloth at the VMSC. A maximum of 10 fish were housed together, receiving a constant flow of aerated, ambient temperature (13–21°C) seawater pumped from Port Phillip Bay and fed frozen bait shrimp twice per week.

### Predator effects on mating behavior

We investigated the effect of predator presence on mating behavior by randomly allocating squid into weight-matched mating pairs (*n* = 15) and then subjecting each pair to three separate predation treatments in a random order. The trials were performed on consecutive days to ensure the females did not lay eggs between trials. The predation treatments were no predator (*no predator*), predator introduced before mating commenced (*predator before*) and predator introduced during mating, one hour after mating commenced (*predator during*). We included these three treatments to investigate whether squid would initiate mating when a predator was present (*predator before*) and whether squid would terminate mating when a predator was introduced near the completion of spermatophore transfer (*predator during*). The latter treatment was necessary because pilot trials suggested that squid may become acclimatized to the threat during the long copulation.

**Figure 2:**
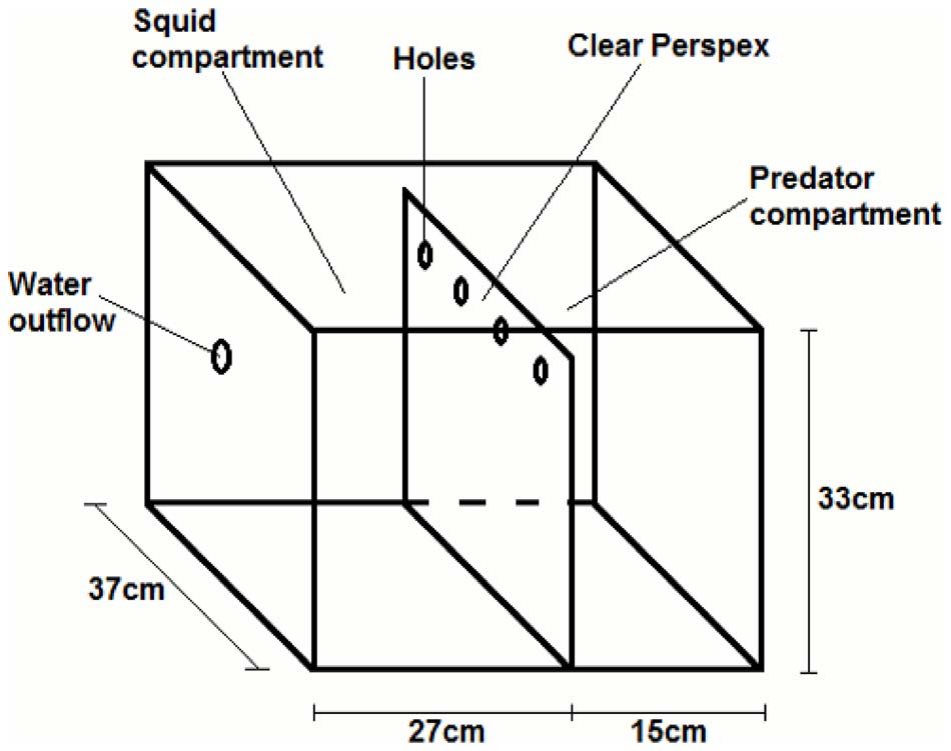
A schematic of the experimental tank. Squid were placed in the left compartment and flathead in the right. Seawater was delivered into the right compartment and would flow out through the outflow in the left compartment. This ensured chemical cues were transferred from flathead to squid.

We conducted trials during the squids' night (i.e. when squid are active). The trials occurred in a tank with two compartments separated by clear Perspex with a row of holes at the top to allow the transmission of olfactory as well as visual cues (squid may be able to detect olfactory cues [40], [41]; Fig. 2). To ensure olfactory cues were transmitted from predator to squid, a constant flow of ambient seawater was delivered into the predator compartment and the outflow was in the squid compartment. There was a 3 cm layer of sand covered the bottom to allow for squid burying behavior. Flathead would not completely bury themselves with this much sand. Each sand flathead was used once in each predation treatment (*predator before* and *predator during*) and was not fed for 3 days before trials.

All trials were recorded using a Sony (North Sydney, Australia) HDR-XR200 camera with night vision function and red lighting to reduce disturbance. A male squid was placed in one compartment and allowed to acclimate for 10 minutes. At 10 minutes either the flathead was introduced into the other compartment using a net (*predator before* treatment) or the net was moved in that compartment as a control (*no predator* and *predator during* treatments). After a further five minutes a female was added and recording began. If mating had not commenced by 10 minutes after recording began, mating was recorded as unsuccessful. However, at this time we gently disturbed the female using a clear plastic rod (*no predator*: *n* = 7; *predator before*: *n* = 9; *predator during*: *n* = 8), causing her to move into the water column, which enabled the male to initiate mating. If mating did not commence within the following five minutes, the trial was terminated (*no predator*: *n* = 2; *predator before*: *n* = 3; *predator during*: *n* = 1). Squid that mated were left for one hour and then either a flathead was introduced (*predator during* treatment) or the net was moved (*no predator* and *predator before* treatments). One pair ceased mating before the predator could be added and thus, was excluded from the analysis of behaviors during and after mating (see below). After this, squid were left to complete mating and the trial was terminated five minutes after the conclusion of mating. We performed all experimental procedures standing behind a black curtain to minimize our influence on squid behavior. From the high definition video footage, we used VLC Media Player (Paris, France) to record the number of jets and inks before and after mating for males and females separately, and the number of jets and inks during mating for the pair combined because it was not possible to distinguish whether it was the male or female inking or jetting during copulation. Inks and jets were converted to frequencies per minute. We also recorded the occurrence of mating (yes/no) and copulation duration (where applicable). Comparisons of before mating variables (inks before, jets before and successful mating) only used data from the *no predator* and *predator before* treatments.

### Ethics Statement

This study was carried out with approval from the University of Melbourne Animal Ethics Committee (ID: 0810874.3) and followed recommendations outlined in Moltschaniwskyj et al. [42]. All animals were collected in Port Phillip Bay, Australia (squid: 38°09′18S, 144°34′03E and 38°10′13S, 144°43′11E; flathead: 38°08′03S, 144°48′30E) under Fisheries Victoria collecting permits (squid: RP962; flathead: RP1019). Neither of the species are currently listed as endangered species and all efforts were made to ameliorate animal suffering.

### Statistical analysis

Residual plots revealed that some behavioral variables required transformation to satisfy the homoscedasticity assumption of a linear model [43]. Due to the large number of zeros in ‘male inks before mating’, ‘female inks before mating’, ‘male inks after mating’ and ‘female inks after mating’ variables, we transformed these data using the fourth root function. For all ‘jet’ variables and the ‘pair inks during mating’ variable, we used a square root transformation as they were not as strongly positively skewed [43]. To test whether squid differed in their behavior in the three treatments, we used repeated measures linear mixed models (PROC MIXED, SAS 9.2). Response variables before mating (female inks, male inks, female jets and male jets) were assessed with treatment (*no predator* and *predator before*), the order in which the treatment was conducted (1^st^, 2^nd^, 3^rd^) and the treatment by treatment order interaction as independent variables. We analyzed inks and jets before mating for each sex separately because males and females experienced different acclimation conditions, thus we cannot directly compare the sexes. All response variables during mating (‘pair inks during mating’ and ‘pair jets during mating’) were assessed with treatment (*no predator*, *predator before*, *predator during*), the order in which the treatment was conducted (1^st^, 2^nd^, 3^rd^) and the treatment by treatment order interaction as independent variables. Response variables after mating (jets and inks) were assessed with treatment (*no predator*, *predator before*, *predator during*), treatment order (1^st^, 2^nd^, 3^rd^), sex (male, female) and all interactions as the independent variables. Treatment was the repeated measure within pairs (during mating variables) or individuals (before and after mating variables) with pair ID or individual ID as the blocking factor. Order was used in all models to account to the repeated use of the same squid pairs and test for any effects on behavior (e.g. habituation, learning). Each model also incorporated the ratio of male to female weight (male weight ÷ female weight +1) as a random covariate. Statistical models were developed in consultation with the University of Melbourne Statistical Consulting Centre.

We performed Fisher's exact tests (PROC FREQ, SAS 9.2) to investigate the effect of treatment (*no predator*, *predator before*) and order (1^st^, 2^nd^, 3^rd^) on occurrence of mating (yes/no; squid that did not mate after 10 minutes were recorded as ‘no’ even if they mated after the female was disturbed). Fisher's exact tests were also performed to assess the effect of treatment (*no predator*, *predator before*, *predator during*) on burying behavior (before and after mating, before only, after only, neither) of males and females.

## Results

**Figure 3:**
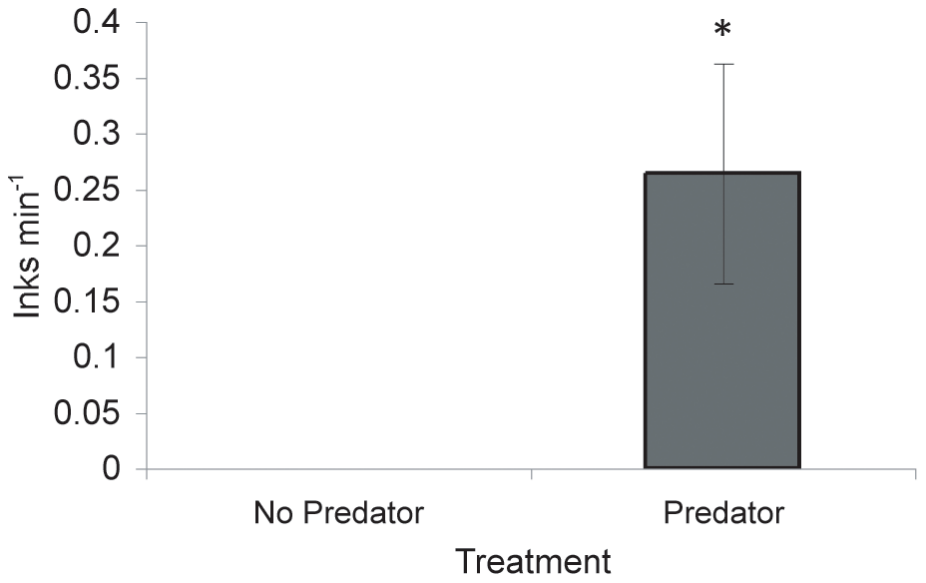
Inks per minute (mean ±SEM) by females before mating commenced in different predation treatments. Asterisk signifies significantly different group (*P*<0.01).

Females inked significantly more when a predator was introduced before mating (*predator before*) than in the absence of a predator (*no predator*; *F*_1, 9_ = 13.73, *P*<0.01; Fig. 3). Other before mating variables (male inks, male jets and female jets) were not affected by treatment or order (Table 1) and there was no effect of predation treatment on burying behavior for either males (Fisher's test; *P* = 0.10) or females (Fisher's test; *P* = 0.51). Males exhibited burying behavior during the acclimation period in 36 out of 42 trials.

**Table 1:** The effect of treatment and order on behaviors before mating for male and female squid.

	Female inks before		Female jets before		Male inks before		Male jets before	
Source	*F _df_*	*p*	*F _df_*	*P*	*F _df_*	*p*	*F _df_*	*p*
Treatment (T)	13.73 _1, 9_	**0.005**	0.05 _1, 9_	0.824	0.37 _1, 9_	0.559	0.05 _1, 9_	0.826
Order (O)	2.57 _2, 9_	0.131	2.44 _2, 9_	0.142	0.37 _2, 9_	0.702	1.05 _2, 9_	0.390
T×O	0.55 _2, 9_	0.597	0.26_ 2, 9_	0.780	0.37 _2, 9_	0.702	0.97 _2, 9_	0.417

Pair inks during mating and pair jets during mating were not affected by treatment (Table 2). There were also no significant effects for the variable inks after mating (Table 3). However, there was a significant interaction between treatment and order for the variable jets after mating (*F*_4, 28_ = 4.47, *P*<0.01). Due to the order effect and to minimize post-hoc comparisons, we assessed the effect of treatment on jets after mating within each order group (1^st^, 2^nd^, 3^rd^ trial) and uncovered no significant differences.

**Table 2:** The effect of order and treatment on behaviors during mating for pairs of squid.

	Duration		Inks During Mating		Jets During Mating	
Source	*F _df_*	*p*	*F _df_*	*p*	*F _df_*	*p*
Treatment (T)	2.36 _2, 20_	0.120	0.26 _2, 14_	0.778	0.56 _2, 14_	0.582
Order (O)	2.45 _2, 20_	0.112	2.15 _2, 14_	0.153	0.51 _2, 14_	0.610
T×O	2.61 _4, 20_	0.066	0.74 _4, 14_	0.581	2.30 _4, 14_	0.110

**Table 3:** The effect of treatment, sex and order on behaviors after mating for individual squid.

	Inks After		Jets After	
Source	*F _df_*	*p*	*F _df_*	*p*
Treatment (T)	0.82 _2, 28_	0.453	0.81 _2, 28_	0.456
Sex (S)	1.56 _1, 28_	0.222	0.67 _1, 28_	0.420
Order (O)	0.42 _2, 28_	0.659	3.75 _2, 28_	**0.036**
T×S	0.75 _2, 28_	0.482	1.01_ 2, 28_	0.377
S×O	2.28 _2, 28_	0.288	0.01 _2, 28_	0.994
O×T	1.32 _4, 28_	0.273	4.47 _4, 28_	**0.006**
T×S×O	1.74 _4, 28_	0.170	0.34 _4, 28_	0.847

Neither treatment nor treatment order influenced the occurrence of successful mating (treatment, Fisher's test; *P* = 0.93; treatment order, Fisher's test; *P* = 0.24) or the duration of copulation (Table 2).

## Discussion

In general, there were few effects of predator presence on mating behavior despite the likelihood that sand flathead is an important predator of dumping squid (sand flathead are common, benthic, ambush predators in Port Phillip Bay). Predation risk did not influence the probability of mating or the duration of copulation and there was no effect of predator presence on anti-predator behaviors during and after mating. However, the strong response of females to predators prior to copulation suggests that flathead are perceived as a significant threat. Specifically, females exhibited a marked increase in inking, a well-documented form of defense in cephalopods [44]–[46]. Inking involves the ejection of a dark cloud of chemicals and is thought to be a visual stimulus which acts like a smokescreen, decoy or distraction [45]. Staudinger et al. [36] have shown that squid that ink have a higher chance of escape when they encounter both ambush and cruising predators. Female dumpling squid are most likely using this form of secondary defense in response to the perceived threat of predation and because they did not have time to bury themselves. This escape response may be an effective way of avoiding mating in the wild where squid are not constrained by an aquarium. Unlike females, males were able to use crypsis as a defense as they received a longer acclimation period than females. During this time, they generally buried themselves in the sand substrate. For cephalopods, the primary form of defense is crypsis [46] and secondary defenses, such as inking, are only employed when individuals perceive an attack to be probable (otherwise inking would give away their position; [36]). It is unlikely that males perceived an imminent attack because burying provides effective camouflage [46]. The general lack of effect of predator presence on mating behavior of the pair, despite its effect on female anti-predator behavior prior to copulation, suggests that dumpling squid may be prioritizing current reproduction.

Despite the female's initial reaction to the predator, there was no effect of predator presence on female inks and jets during or after mating. Since females reacted to a predator before mating commenced, we can be confident that squid were aware of the addition of a predator during mating. Inking and jetting are likely to be energetically costly behaviors and may be less effective during mating (i.e. jetting is likely to be slower and uncoordinated with two individuals). In addition, it is probable that females are unable to ink or jet during mating due to the copulatory grip (Fig. 1), which visibly constricts the female's mantle cavity. Mating has a significant energetic cost: both sexes have reduced swimming endurance after mating which lasts for up to 30 minutes [34]. This energetic cost may compromise the squid's ability to ink and jet during copulation and immediately afterwards (video recordings of behavior were stopped five minutes after mating had ceased).

Our data further suggest that squid are likely to copulate even under predation risk as the occurrence of successful mating and copulation duration were not influenced by predator presence. Males, which initiate copulation and appear to control its duration, were undeterred by the perceived predation risk and the increase in inking by females. A lack of change in the probability of copulation with increasing predation risk is also observed in other species [24], [25]. For example, male wolf spiders do not alter courtship intensity or duration, or experience a decrease in mating probability under increased predation. However, they may experience lower mating success in these conditions because there are more failed palpal insertions [26]. Alternatively, predation risk may not affect the probability of mating if there is a decrease in movement associated with mating that reduces conspicuousness to a predator [24]. This is unlikely in *Euprymna tasmanica* because they seem to be more conspicuous during mating (they are in the open and no longer buried in the sand).

Males also did not terminate copulations early in the presence of a predator. This is similar to our observations of mating pairs during fieldwork. Our close proximity to mating pairs never caused copulation to cease and captured mating pairs would continue copulating throughout transport to the laboratory which could take up to one hour. Males appear to require the full mating duration to successfully transfer and evert all spermatophores (pers. obs.). Sperm competition is predicted to be a central feature of cephalopod mating systems because females are polyandrous and can store sperm for prolonged periods [29]. If the male were to terminate copulation early he may transfer and evert fewer spermatophores, which is likely to directly reduce his share of fertilizations. There is evidence in many taxa that reduced copulation duration decreases fertilization success [22], [47], [48]. Thus, the benefits of completing mating may outweigh the risk of predation if this risk is perceived to be low.

Overall, dumpling squid may show little change in mating behavior in response to the presence of sand flathead because the benefits of mating outweigh the perceived risk of predation. *Euprymna tasmanica* is a solitary species with a short reproductive lifespan, thus, it may be important for them to secure mating opportunities whenever they arise. Furthermore, it may be beneficial to continue mating once started because of the high energetic investment into extended copulation in this species [34]. This energetic investment could affect the motivation to end mating in the presence of a predator, particularly near the end of mating when squid may be too physically exhausted to escape. However, we found no difference in the propensity to terminate mating when the predator was introduced prior to or one hour after mating commenced. Nevertheless, the perceived risk of predation may not have been high enough to outweigh the benefits of completing mating. To understand the fitness effects of prioritizing current reproduction over future reproduction, more data quantifying mating opportunities and mating frequencies in the wild are required.

## Conclusions

The mating behavior of short-lived and/or solitary species is likely to be strongly influenced by their chance of future reproduction [3], [15]. For example, the antechinus, a small, carnivorous marsupial, has a semelparous reproductive strategy with extreme terminal investment because the few females that survive until their second breeding season do not have greater reproductive success [49]. In our study, whilst females increased their anti-predator behaviour in response to predation risk, this risk did not deter pairs from mating or reduce the copulation duration suggesting that dumpling squid are prioritising current reproduction under these conditions. Prioritizing current reproduction, even under predation risk, is expected to be adaptive when the chance of future reproduction is low (such as for short-lived, solitary taxa, or older individuals) and when the potential fitness payoffs of mating are high (such as for species where multiple mating has a substantial benefit). Thus, to understand how predation may affect mating behavior, it is essential to consider both the species ecology and life history.

## Supporting Information

S1 Table **The duration of sexual maturity in** ***Euprymna tasmanica.*** Data from 29 dumpling squid housed in the laboratory at the Victorian Marine Science Consortium, Queenscliff. Sexual maturity was recorded as the day that the nidamental gland was visible through the translucent ventral wall [1].
(DOCX)

S1 Video **Squid behaviors: Inking, jetting and copulation.** This video depicts four squid behaviors. Start of Copulation: Male is buried in the sand and grabs female. Female inks as she is grabbed. Inking: Squid in top right corner inks just before clip cuts. Jetting: Squid at top of the screen is jetting. End of Copulation: Male (the darker squid) forcefully disentangles himself from the female.
(MP4)
