# Supplementary material for: Does Predation Risk Affect Mating Behavior? An Experimental Test in Dumpling Squid (Euprymna tasmanica)
Source: PLoS One. 2014 Dec 31;9(12):e115027. doi: 10.1371/journal.pone.0115027 (PMC4281177; doi:10.1371/journal.pone.0115027)
Supplement: S1 Table — The duration of sexual maturity in Euprymna tasmanica. Data from 29 dumpling squid housed in the laboratory at the Victorian Marine Science Consortium, Queenscliff. Sexual maturity was recorded as the day that the nidamental gland was visible through the translucent ventral wall [1]. (DOCX) [file pone.0115027.s001.docx]

**Does predation risk affect mating behavior? An experimental test in dumpling squid (*Euprymna tasmanica*) – Supporting information**

Amanda M. Franklin, Zoe E. Squires & Devi Stuart-Fox

**Table S1: The duration of sexual maturity in *Euprymna tasmanica*.** Data from 29 dumpling squid housed in the laboratory at the Victorian Marine Science Consortium, Queenscliff. Sexual maturity was recorded as the day that the nidamental gland was visible through the translucent ventral wall [1].

|  | **ID** | **Sexual Maturity (day #)** | **Died (day #)** | **Duration of Sexual Maturity (days)** |
| --- | --- | --- | --- | --- |
|  | 168 | 482 | 548 | 66 |
|  | 169 | 482 | 543 | 61 |
|  | 177 | 490 | 574 | 84 |
|  | 181 | 493 | 560 | 67 |
|  | 183 | 493 | 562 | 69 |
|  | 186 | 495 | 543 | 48 |
|  | 187 | 499 | 617 | 118 |
|  | 189 | 508 | 590 | 82 |
|  | 191 | 511 | 594 | 83 |
|  | 192 | 512 | 569 | 57 |
|  | 195 | 531 | 594 | 63 |
|  | 196 | 541 | 590 | 49 |
|  | 197 | 546 | 606 | 60 |
|  | 198 | 549 | 608 | 59 |
|  | 199 | 549 | 597 | 48 |
|  | 209 | 560 | 611 | 51 |
|  | 210 | 560 | 617 | 57 |
|  | 211 | 560 | 612 | 52 |
|  | 212 | 563 | 679 | 116 |
|  | 213 | 564 | 634 | 70 |
|  | 218 | 578 | 634 | 56 |
|  | 219 | 578 | 688 | 110 |
|  | 220 | 578 | 735 | 157 |
|  | 222 | 580 | 658 | 78 |
|  | 232 | 586 | 639 | 53 |
|  | 228 | 592 | 722 | 130 |
|  | 229 | 594 | 718 | 124 |
|  | 230 | 594 | 711 | 117 |
|  | 231 | 594 | 715 | 121 |
| Average |  |  |  | 80 |
| Standard Deviation |  |  |  | 31 |
| SEM |  |  |  | 5.68 |

**Supporting Information References**

1. Bloodgood RA (1977) The squid accessory nidamental gland: Ultrastructure and association with bacteria. Tissue Cell 9: 197-208.
